# Supplementary material for: MetaRibo-Seq measures translation in microbiomes
Source: Nat Commun. 2020 Jun 29;11:3268. doi: 10.1038/s41467-020-17081-z (PMC7324362; doi:10.1038/s41467-020-17081-z)
Supplement: Supplementary file 10 — Supplementary Data 7 [file 41467_2020_17081_MOESM10_ESM.zip › File2/Confidence_High_Taxonomy/206461_out.krona.html]

Javascript must be enabled to view this page.

members
magnitude
magnitudeUnassigned
count
unassigned
taxon
rank

206461\_out

5

superkingdom
2
5

5
1239
phylum

186801
5
class

5
186802
order

4
186806
family

4

SRS019068\_contig\_number\_95257SRS104636\_contig\_number\_contig-100\_41159.41160
1730
2
genus

species

SRS149879\_contig\_number\_contig-100\_9799.59714
1897002
1

species

SRS148159\_contig\_number\_21601
1263078
1

186803
1
family

genus
1
2569097

39488

SRS144183\_contig\_number\_28977
1
species
